# Hyaluronic acid ameliorates intervertebral disc degeneration *via* promoting mitophagy activation

**DOI:** 10.3389/fbioe.2022.1057429

**Published:** 2022-12-16

**Authors:** Fu Zhang, Songjuan Wang, Manman Gao, Baoliang Li, Zhongyuan He, Tao Tang, Zhengya Zhu, Shaoyu Liu, Zhiyu Zhou

**Affiliations:** ^1^ Innovation Platform of Regeneration and Repair of Spinal Cord and Nerve Injury, Department of Orthopaedic Surgery, The Seventh Affiliated Hospital of Sun Yat-sen University, Shenzhen, China; ^2^ Department of Orthopaedic Surgery, Shenzhen Second People’s Hospital, The First Affiliated Hospital of Shenzhen University, Shenzhen, China; ^3^ Department of Medical Ultrasonic, The Seventh Affiliated Hospital of Sun Yat-sen University, Shenzhen, China; ^4^ Guangdong Provincial Key Laboratory of Orthopedics and Traumatology, The First Affiliated Hospital of Sun Yat-sen University, Guangzhou, China

**Keywords:** intervertebral disc degeneration, hyaluronic acid, mitophagy, oxidative stress, C1QBP

## Abstract

Activation of mitophagy was considered to be a potential therapeutic strategy for intervertebral disc degeneration (IDD). There was evidence suggesting that hyaluronic acid (HA) can protect mitochondria from oxidative stress in chondrocytes, but its protective effects and mechanism in nucleus pulposus cells (NPCs) remain unclear. This study aimed to confirm the effect of HA promoting mitophagy and protecting mitochondria function in NPCs, and explore its underlying mechanism. NPCs were treated with high molecular weight HA, tert-butyl hydroperoxide (TBHP) and Cyclosporin A (CsA). Mitophagy, mitochondrial function, apoptosis, senescence and extracellular matrix (ECM) degradation were measured. Then, NPCs were transfected with C1QBP siRNA, mitophagy and mitochondrial function were tested. The therapeutic effects of HA on IDD by promoting mitophagy were assessed in bovine intervertebral disc organ culture model. The results showed that TBHP induced oxidative stress, mitochondrial dysfunction, NPCs apoptosis, senescence and ECM degradation. Treated by HA, mitophagy was activated, concomitantly, mitochondrial dysfunction, apoptosis, senescence and ECM degradation were ameliorated. Mitophagy inhibition by CsA partially eliminated the protective effects of HA against oxidative stress. After transfected with C1QBP siRNA to reduce the expression of C1QBP in NPCs, the effect of HA promoting mitophagy was inhibited and the protective effect of HA against oxidative stress was weaken. Additionally, HA alleviated NPCs apoptosis and ECM degradation in bovine intervertebral disc organ culture model. These findings suggest that HA can protect mitochondrial function through activation of mitophagy in NPCs and ameliorate IDD. Furthermore, C1QBP is involved in HA promoting mitophagy and protecting NPCs from oxidative stress. Taken together, our results provide substantial evidence for the clinical applications of HA in the prevention and treatment of IDD.

## Introduction

Low back pain (LBP) is a complicated and prevalent musculoskeletal disorder that affects up to 80% of the population during their lifetime, imposing enormous health and economic burdens on society (Fitzmaurice et al., 2018; Ravindra et al., 2018). Intervertebral disc degeneration (IDD) is widely acknowledged to be a major causative factor of LBP (Risbud et al., 2014; Vlaeyen et al., 2018). However, until very recently, all therapies for IDD have limited efficacy, because they can’t treat the underlying degeneration or restore the native function of intervertebral disc (IVD) (Deyo et al., 2016).

The etiology of IDD is multifactorial, but the increasing level of reactive oxygen species (ROS) caused by several pathological factors such as mechanical stress, inflammatory cytokines, nutrition deprivation, and senescence, plays a crucial role in the occurrence and progression of IDD (Feng et al., 2017). Mitochondria is not only the main source of ROS, but also vulnerable to oxidative injury (Ni et al., 2015). The overproduction of ROS furtherly provokes oxidative stress, resulting mitochondrial dysfunction and activating endoplasmic reticulum stress, which terminally contributes to trigger a cascade of apoptosis and cell death (Wang et al., 2018). Hence, targeting the oxidative stress induced by mitochondrial dysfunction of nucleus pulposus cells (NPCs) might be a potential therapeutic strategy for IDD.

Mitophagy is a special type of autophagy that timely and selectively removes the damaged mitochondria, which is important for maintaining mitochondrial homoeostasis and quality control (Pickles et al., 2018). The basic level of mitophagy maintains mitochondrial homeostasis and protects cells. Under the influence of several cellular stress including oxidative stress and nutrition deprivation, mitophagy can be activated to promote cell survival by eliminating dysfunctional mitochondria (Kang et al., 2020; Zhang et al., 2018). Recently, an increasing studies have identified that mitophagy plays an important role in slowing the pathological process of several diseases, such as neurodegenerative diseases (Lou et al., 2020), kidney diseases (Livingston et al., 2019), and joint degenerative diseases (Sun et al., 2020). Therefore, activation of mitophagy might be a potential therapeutic strategy for protecting NPCs from oxidative stress and slowing the process of IDD.

Hyaluronic acid (HA), a natural disaccharide polymer, is the major component of extracellular matrix (ECM). HA has been studied in several diseases, especially in osteoarthritis and IDD, due to its following physical properties: excellent viscoelasticity, high biocompatibility, and moisture retention capacity (Vasvani et al., 2020). In addition, with the more pharmacological effects discovered, such as: antioxidative, anti-inflammatory, analgesic, anti-apoptosis and inhibiting ECM degradation, HA has become more widely accepted in the treatment of joint degenerative diseases (Cooper et al., 2017; Gupta et al., 2019; Sloan et al., 2020). Moreover, there was evidence suggesting that HA can prevent mitochondrial dysfunction and mitochondria-driven apoptosis on chondrocytes, which was essential for its chondroprotective action (Grishko et al., 2009). The protein C1QBP (complement component 1q binding protein), as hyaluronic acid-binding protein (HABP1), has been reported that played an important role in mitochondrial quality control and mitophagy regulation under physiological condition (Jiao et al., 2015). Based on these above findings, we put forward the hypothesis that HA can protect mitochondrial function through activation of mitophagy in NPCs, and ameliorate IDD.

Therefore, in this study, we studied the effect of HA promoting mitophagy under oxidative stress in NPCs, and explored its underlying mechanism. Furthermore, we evaluated the therapeutic potential of HA in a bovine IVD organ culture model.

## Materials and methods

### Human NPCs isolation and culture

Nucleus pulposus (NP) tissue was obtained during the discectomy from 8 patients undergoing lumbar degenerative diseases (4 female, 4 male, age 46–65 years) (Table 1). To exclude any contamination between annulus fibrosus and NP region, only the innermost part of the disc was collected as NP tissue (Feng et al., 2013), and this process was performed under the microscope (Chen et al., 2016). All procedures were conducted in accordance with the guidance and approval of the Research Ethics Committee of the Seventh Affiliated Hospital of Sun Yat-sen University. To investigate the relationship between C1QBP and the degree of disc degeneration, 6 NP tissues were used for Real Time Quantitative PCR (RT-qPCR) experiment and divided into two groups according to the Pfirrmann grading system (Pfirrmann et al., 2001): the mild degeneration group (II -III grade, *n* = 3) and the severe degeneration group (IV-V grade, *n* = 3).

**TABLE 1:** Clinical patient characteristics.

Patient	Age	Gender	Disc level	Degeneration grade	Experiment
1	65	female	L5/S1	III	Cell culture
2	63	male	L4/5	III	Cell culture
3	46	female	L4/5	III	Cell culture, RT-qPCR
4	47	female	L4/5	III	Cell culture, RT-qPCR
5	46	male	L4/5	V	RT-qPCR
6	53	female	L5/S1	II	RT-qPCR
7	63	male	L3/4	IV	RT-qPCR
8	47	male	L5/S1	IV	RT-qPCR

The tissue in the central part of NP was washed three times with phosphate buffered saline (PBS, Gibco, United States) to remove blood, and then cut into 1 mm^3^ pieces. Subsequently, the NP tissue was treated by 0.25% trypsin-EDTA (Gibco) for 30 min, then digested with 0.25% Collagenase II (Sigma-Aldrich, United States) at 37°C for 4 h. After filtered by 70-micron nylon mesh and separated by centrifugation at 1000 g for 4 min, the NPCs were cultured in Dulbecco’s Modified Eagle’s Medium (DMEM)/Nutrition Mix F12 (Gibco) containing 10% Fetal Bovine Serum (FBS, Gibco) and 1% penicillin/streptomycin (Gibco) at the humidified atmosphere with 5% CO_2_ at 37°C.

### Isolation and cultivation of IVDs

Bovine caudal IVDs were isolated and cultivated as previously described (Dudli et al., 2014). In brief, individual IVDs with endplates were harvested with a band saw after removal of the surrounding soft tissue. The Endplates were rinsed with PBS using an APEXPULSE Disposable Pulse Lavage system (Apex, Guangzhou, China). Then, the IVDs were incubated in PBS containing 10% penicillin/streptomycin (Gibco) for 15 min. Subsequently, IVDs were cultured in DMEM containing 2% fetal calf serum (FCS), 1% penicillin/streptomycin, 50 μg/ml l-ascorbic acid (Sigma-Aldrich), 1% ITS + Premix (Sigma-Aldrich) and 0.1% Primocin (InvivoGen, United States) at the humidified atmosphere (85%) with 5% CO_2_ at 37°C. From day 1, the medium was changed once a day after the IVDs were cultured in our custom designed bioreactor under physiological loading (0.02–0.2 MPa; 0.2 Hz; 1 h/day) (Lang et al., 2018; Zhou et al., 2021).

### Experimental design

NPCs: To explore the optimal concentration and time of tert-butyl hydroperoxide (TBHP) inducing oxidative stress, NPCs were treated with different concentrations of TBHP (Sigma-Aldrich) (0, 25, 50, 100, 200 and 400 µM) for 12 h and with the same concentration of TBHP (200 μM) for different times (0, 0.5, 1, 2, 4 and 6 h). We found that NPCs viability was significantly reduced after treated by 200 µM TBHP for 2 h. Therefore, in the subsequent experiments, the NPCs were treated by 200 µM TBHP for 2 h, to induce oxidative stress. High molecular weight (MW)-HA (MW = 1800–2200 kDa) was purchased from Bloomage Biotechnology (Jinan, China), and it was produced by bacterial fermentation. NPCs were treated with HA for 24 h prior to administration of TBHP, to explore the protective effect of HA in NPCs. The concentration of HA was 1 µM (Rayahin et al., 2015). Cyclosporin A (CsA, MCE, United States) (1 μM), a mitophagy inhibitor (Lin et al., 2020), was used for 1 h before HA administration, to confirm the protective effect of mitophagy activation by HA.

IVDs: Mechanical stress (MS) was induced by one strike loading as previously described (Li et al., 2021). Briefly, IVDs were incubated in a custom designed incubation chamber on day 2. After preload of 10 N for 3 min to ensure that the load cells were in contact with the IVDs, MS injury was applied using a custom-made universal mechanical tester by compression to 40% of disc height in 1 s. All IVDs were randomly assigned to four groups: Control Group, MS Group, MS + HA Group, MS + HA + CsA Group. Control Group was received only physiological loading. MS Group was received one strike loading. MS + HA Group: 100 μL PBS containing 1 µM high MW-HA was injected into IVDs for 2 h before MS injury. MS + HA + CsA Group:100 µL PBS containing 1 µM high MW-HA and 1 µM CsA was injected into IVDs for 2 h before MS injury, to confirm the protective effect of mitophagy activation by HA in IVDs. After loading, the IVDs were incubated in a culture medium with daily physiological loading for another 3 days, and the medium was changed once a day.

### Cell viability assay

The Cell Counting Kit-8 (CCK-8, MCE) were used to evaluate cell viability. NPCs were seeded in 96-well plates (5 × 10^3^ cells/well) and incubated in complete medium for 24 h. Following treated by TBHP, 10 µL CCK-8 solution was added to cells, and incubated at 37°C for 2 h. Finally, the well absorbance was monitored spectrophotometrically at 450 nm.

### Western blot assay

The total protein was obtained by lysing NPCs with Radio Immunoprecipitation Assay (RIPA) Lysis Buffer (Boster, Wuhan, China) containing 1% Phenylmethylsulfonyl fluoride (PMSF, Boster), and protein concentration was detected by the BCA protein assay kit (Boster). The total protein was separated by electrophoresis on NuPAGE™ 4%–12% Bis-Tris gels (Invitrogen) and transferred to a polyvinylidene fluoride (PVDF) membrane (Invitrogen). After blocked with 5% nonfat dry milk in TBST (Leagene, Beijing, China) for 2 h and washed 3 times with TBST, the protein bands were incubated with primary antibodies: Beclin-1 (1:1000, Cell Signaling Technology, United States), LC3 (1:1000, Cell Signaling Technology), Bax (1:1000, Proteintech, China), Bcl2 (1:1000, Abcam), Mmp13 (1:1000, Abcam), C1QBP (1:1000, Cell Signaling Technology), P62 (1:500, Cell Signaling Technology) and β-actin (1:1000, Cell Signaling Technology) at 4°C overnight. After washing three times with TBST, the protein bands were incubated with goat anti-rabbit horseradish peroxidase (HRP) (1:4000, Abcam) or goat anti-mouse HRP (1:4000, Abcam) secondary antibody for 2 h at room temperature. Ultimately, after washing three times with TBST, the band was treated by enhanced chemiluminescence (ECL) reagent (Beyotime, Shanghai, China). Quantitative analysis for the protein band densities was performed using ImageJ software (National Institutes of Health, United States) and presented as relative level to β-actin.

### RT-qPCR

Total RNA was extracted using RNAeasy™ Animal RNA Isolation Kit (Beyotime) according to the manufacturer’s instruction. Subsequently, 500 ug total RNA was used to synthesize cDNA using PrimeScript™ RT Master Mix (Thermo Fisher Scientific, United States). RT-qPCR was performed using PowerUp™ SYBR™ Green Master Mix (Applied Biosystems, United States) according to the manufacturer’s protocol, in CFX96 Real-Time System (Bio-Rad Laboratories, United States). The total volume of each PCR reaction is 10 μL, including 5 μL SYBR Green Master Mix (2 x), 2 µL RNase-free dH2O, 0.5 µL of each primer (Table 2) and 2 µL diluted cDNA. The results were normalized to the GAPDH level and evaluated by using the 2^−ΔΔCt^ method (Schmittgen et al., 2008).

**TABLE 2:** Primer sequences used for RT-qPCR experiments.

Gene	Organism	Sense primer	Antisense primer
C1QBP	*Homo sapiens*	GGA​CAA​GAA​GAC​GAG​GCT​GAG​AGT	CGG​TCG​GCA​AGG​AAA​TCC​ATT​AGG
Aggrecan	*Homo sapiens*	TCC​CCT​GCT​ATT​TCA​TCG​AC	CCA​GCA​GCA​CTA​CCT​CCT​TC
Collagen II	*Homo sapiens*	GGA​GCA​GCA​AGA​GCA​AGG​AGA​A	AGC​AGG​CGT​AGG​AAG​GTC​ATC​T
GAPDH	*Homo sapiens*	CGG​AGC​CAA​AAG​GGT​CAT​CA	GGG​GGG​CTA​AGC​AGT​TGG​TG

### Immunofluorescence (IF) staining

NPCs were fixed on coverslips with 4% paraformaldehyde for 20 min, then permeabilized and blocked with PBS containing 0.3% Triton X-100 and 5% BSA for 1 h at room temperature. The slides were then incubated with primary antibodies collagen II (1:200, Cell Signaling Technology), Aggrecan (1:200, Abcam), Tom20 (1:200, Cell Signaling Technology), LC3 (1:200, Cell Signaling Technology) at 4°C overnight. After washing with TBST, slides were incubated with Alexa Fluor-488 or Fluor-594 conjugated secondary antibody (1:300, Jackson ImmunoResearch Inc., United States) for 1 h at room temperature and labeled with 4′,6-Diamidino-2-Phenylindole (DAPI, Abcam) for 5 min. Finally, the staining was visualized in a confocal fluorescence microscope (Zeiss Confocal LSM 780, Germany) and fluorescence intensity was measured using ImageJ software.

Bovine caudal NP tissues were fixed in 4% paraformaldehyde for 24 h and dehydrated in graded sucrose solutions. Then, the tissues were embedded in OCT compound (Sakura, United States), and cut into 10 µm-thick cryosections. The following permeabilization, block and immunostaining protocols were identical to coverslips as described above. These following primary antibodies were used: Bax (1:200, Proteintech), collagen II (1:200, Affinity, United States), Mmp13 (1:200, Proteintech).

### Mitochondrial membrane potential (Δψm) assay

The Δψm was assayed using a the mitochondrial-specific fluorescent probe JC-1 (5,5′,6,6′-Tetrachloro-1,1′,3,3′-tetraethyl-imidacarbocyanine, MCE) according to the manufacturer’s protocol. Brief, NPCs were incubated with JC-1 solution (10 μM) for 30 min at 37°C, and washed three times with PBS. The staining was observed using a fluorescence microscope (Leica DM6B, Germany).

### Measurement of mitochondrial superoxide

Mitochondrial Superoxide was measured by using MitoSOX™ Red Superoxide Indicator (Invitrogen). After treatment, NPCs were incubated with 5 μM MitoSOX™ reagent working solution for 15 min at 37°C, then washed with PBS and observed by a fluorescence microscope.

### Reactive oxygen species assay

Dihydroethidium (DHE) staining was used to test the production of ROS. NPCs were seeded on glass coverslips in complete culture medium. After treatment, the cells were incubated with 10 µM DHE solution (MCE), then washed with PBS and observed by a fluorescence microscope.

### ATP assay test

The ATP level was assayed using ATP Assay Kit (Beyotime) according to the manufacturer’s protocol. After treatment, NPCs were lysed and centrifugated at 12000 g for 5 min at 4°C. Then, the samples were treated by 100 µL ATP assay solution and the relative light unit was monitored by luminometer.

### TUNEL assay

To detect apoptosis of NPCs, apoptotic DNA fragmentations were measured by TdT-mediated dUTP Nick End Labeling (TUNEL) staining (Meilunbio, Dalian, China) according to the manufacturer’s instruction. In brief, NPCs were fixed with 4% paraformaldehyde for 30 min, then permeabilized with PBS containing 0.3% Triton X-100 for 5 min at room temperature. After washed three times with PBS, the cells were stained with TUNEL reagent working solution containing TdT Enzyme (10x) and FITC-12-dUTP Labeling Mix for 1 h at 37°C, protected from light. Finally, the image was observed using a fluorescence microscope.

### SA-β-gal staining

SA-β-gal staining kit (Beyotime) was used to detect the degree of senescence of NPCs according to the manufacturer’s protocol. After being washed twice times with PBS, NPCs on plates were fixed with 0.2% paraformaldehyde for 20 min at room temperature. Subsequently, the cells were stained with X-gal staining solution overnight at 37°C. Images were captured using optical microscopy (Olympus, Japan) and the percentages of SA-β-gal-positive cells were quantified for statistical analysis.

### siRNA Transfections

siRNA for C1QBP was designed and synthesized by Transheep Biotechnology (Shanghai, China). The NPCs were transfected with C1QBP siRNA or control siRNA using Namipo™ transfection reagent according to the manufacturer’s protocol. When NPCs were at a confluence of 50%–60%, C1QBP siRNA or control siRNA was diluted in Namipo™ transfection reagent and added to cells. Following incubation for 2 days, the transfected cells were treated by HA and TBHP, and the mitochondrial function was measured by JC-1 staining or MitoSox Red staining. Meanwhile, the expression of C1QBP, P62 and Beclin-1 was quantified by Western blot analysis.

### Statistical analysis

Statistical analyses were conducted using SPSS 22.0 (IBM, United States). The Shapiro–Wilk normality test was performed to checked the data distribution. Comparisons between two groups were performed by Student’s t-test for normally distributed data and Mann–Whitney test for non-normally distributed data. Multiple comparisons of data among the groups were analyzed by one-way analysis of variance (ANOVA) followed by Tukey’s test for comparison between the two groups. Non-parametric data were analyzed by the Kruskal–Wallis H test followed by Dunn’s test with Bonferroni corrections. *p* < 0.05 was considered statistically significant.

## Results

### HA Induces Mitophagy in NPCs

**FIGURE 1:**
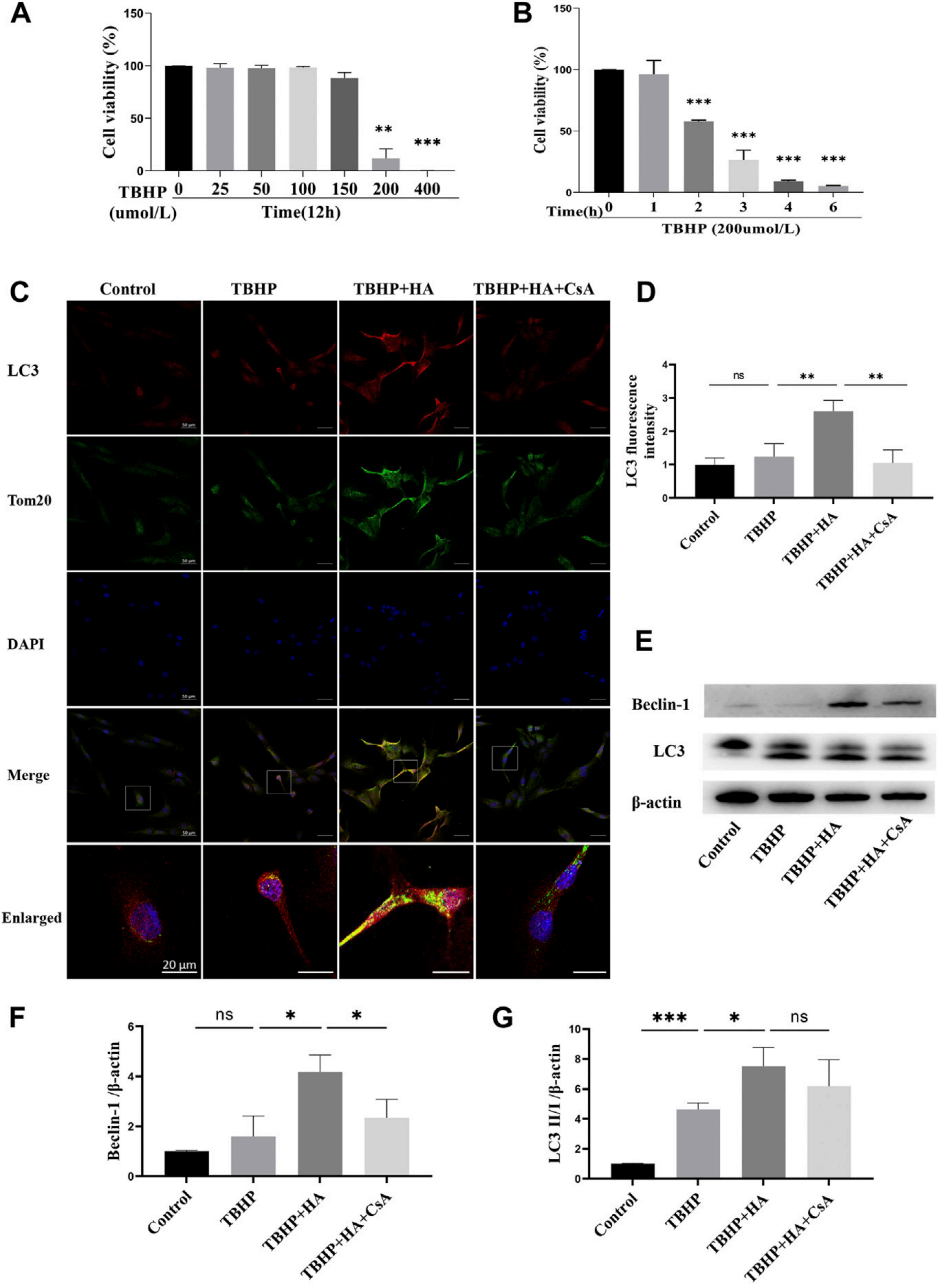
HA Induces Mitophagy in NPCs. **(A,B)** Effects of TBHP on cell viability detected by CCK-8.**(C,D)** The colocalization of LC3 and Tom20 was examined by confocal microscopy. LC3 (red), Tom20 (green), DAPI (blue). **(E–G)** The protein expression of Beclin-1 and LC3 was tested by Western blot. Data are represented as the mean ± SD. ns, not significant, *p < 0.05, **p < 0.01, ***p < 0.001. Scale bar: 50 or 20 μm *n* = 3.

In order to explore the optimal condition for TBHP to induce oxidative stress, NPCs were treated by TBHP with different concentration or time, and cell viability was detected by cck-8 assay. We found that NPCs viability was significantly reduced after treated by 200 µM TBHP for 2 h (Figures 1A,B). The immunofluorescence staining results showed that co-localization of autophagosome formation marker LC3 on mitochondria was significantly increased in the HA-treated group, which evidenced the formation of mitophagosomes. However, after pretreatment with mitophagy inhibitor CsA, the co-localization fluorescence signal of LC3 and Tom20 was rapidly decreased (Figures 1C,D). Consistent with this, Western blot analysis also revealed that the protein expression of LC3 II/I and Beclin-1 was increased in NPCs treated with HA, and decreased after CsA treatment (Figures 1E–G). Therefore, these data indicated that HA promoted mitophagy under oxidative stress, which can be inhibited by mitophagy inhibitor CsA.

### HA attenuates TBHP-induced mitochondrial dysfunction in NPCs

**FIGURE 2:**
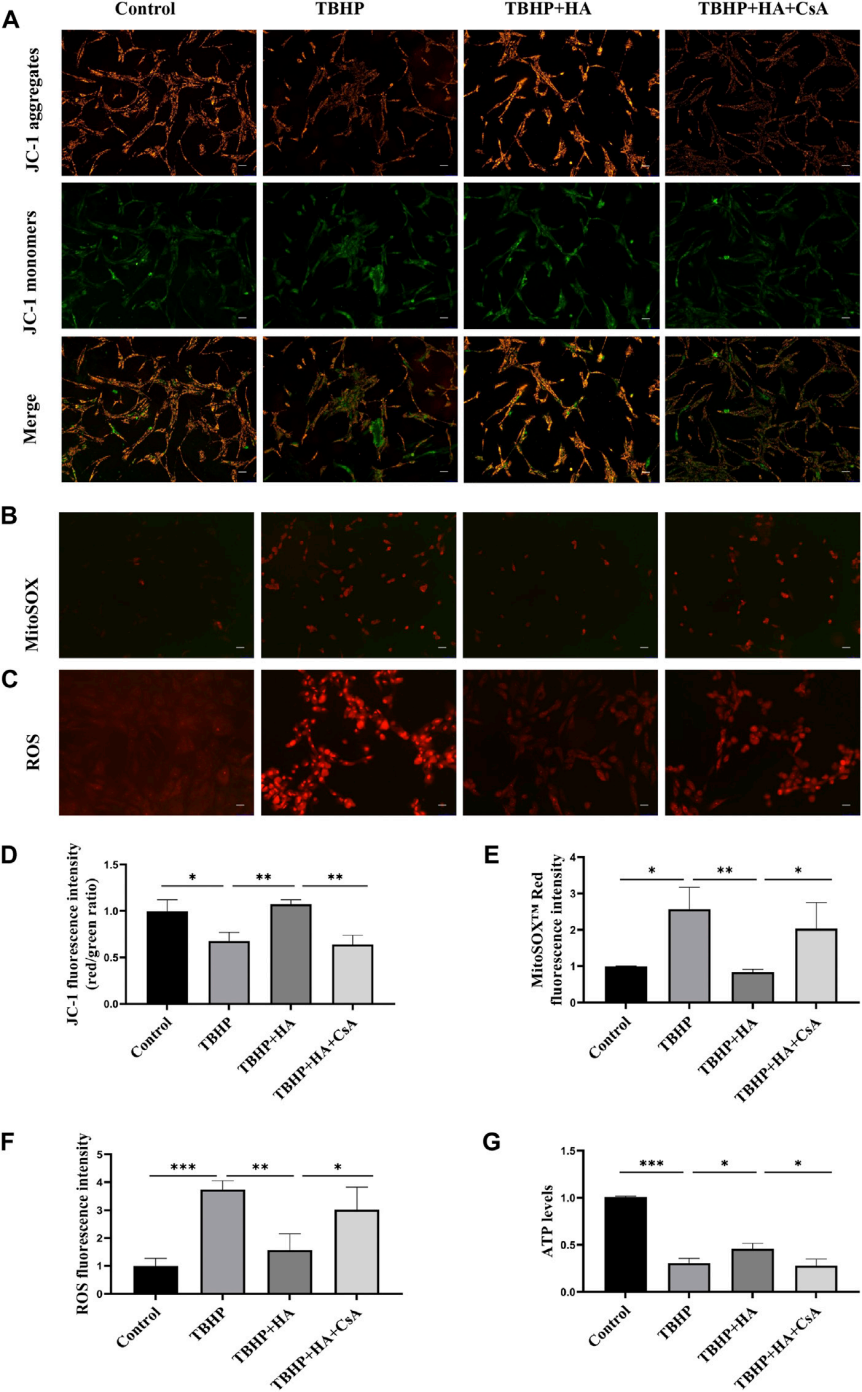
HA attenuates TBHP-induced mitochondrial dysfunction in NPCs. **(A)** Δψm in NPCs was measured by JC-1 staining. **(B)** The production of mitochondrial Superoxide was measured by using MitoSOX™ Red Superoxide Indicator. **(C)** The production of ROS was tested by DHE staining. **(D–F)** The relative fluorescence intensity was measured using ImageJ software. **(G)** Cellular ATP levels of NPCs were measured. Data are represented as the mean ± SD. *p < 0.05, **p < 0.01, ***p < 0.001. Scale bar: 50 μm *n* = 3.

To investigate whether HA attenuates TBHP-induced mitochondrial dysfunction by mitophagy, NPCs were pretreated with mitophagy inhibitor CsA before HA administration. The JC-1 staining was used to test Δψm, the MitoSox Red staining was used to test Superoxide produced by mitochondrial, and DHE staining was used to test ROS produced by NPCs. First, NPCs were treated with TBHP, the Δψm of NPCs was reduced. While the Δψm was recovered by HA administration compared to the TBHP-treatment group, the preservation of Δψm failed while the cells were pretreated with CsA (Figures 2A,D). Second, increased superoxide production induced by TBHP was attenuated by HA treatment, CsA pretreatment was observed to reverse these effects (Figures 2B,E). Third, the change of intracellular ROS production was consistent with the superoxide results (Figures 2C,F). Finally, cellular ATP level was decreased in TBHP treatment group. After pretreatment with HA, the cellular ATP level in NPCs increased markedly compared with that of the TBHP-treated group, but CsA pretreatment can eliminate of the protective effect of HA (Figure 2G). These results indicated that HA attenuates TBHP-induced mitochondrial dysfunction. However, inhibition of mitophagy by CsA pretreatment can block the protective effects of HA.

### HA attenuates TBHP-induced apoptosis and senescence in NPCs

**FIGURE 3:**
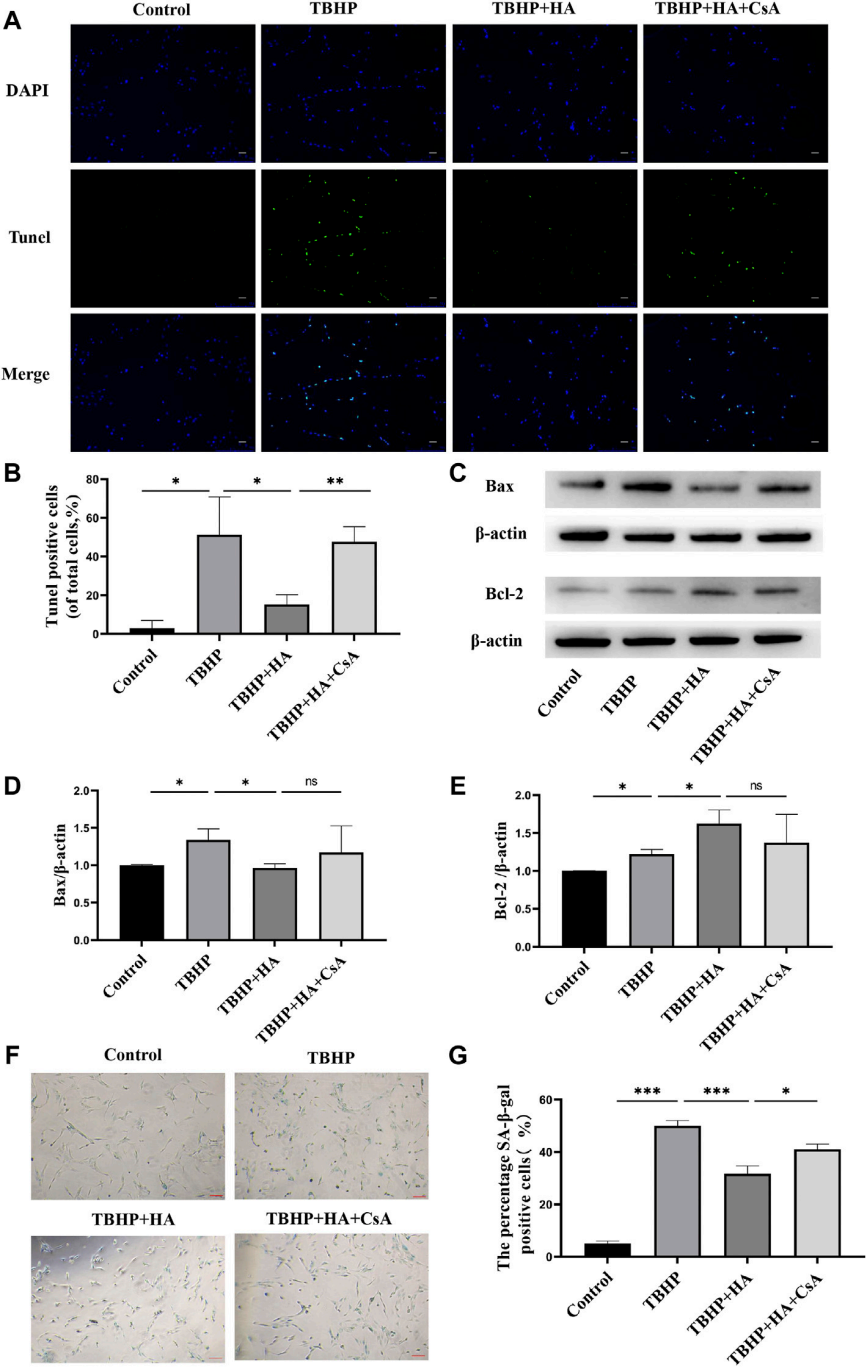
HA attenuates TBHP-induced apoptosis and senescence in NPCs. **(A,B)** TUNEL assay was performed in NPCs as treated above. Scale bar: 50 μm. **(C–E)** Western blotting was performed to detect the expression of apoptosis-related proteins Bax and Bcl-2 in NPCs. **(F,G)** Representative images of SA-β-gal staining of NPCs. Scale bar: 100 μM.Data are represented as the mean ± SD. ns, not significant, *p < 0.05, **p < 0.01, ***p < 0.001. *n* = 3.

The TUNEL assay results demonstrated that the apoptotic cells were increased in TBHP treatment group, and HA was observed to reverse TBHP-induced cell apoptosis. Inhibition of mitophagy by CsA pretreatment can eliminate of the protective effect of HA (Figures 3A,B). In addition, the results of the Western blot analysis indicated the effects of HA on reducing pro-apoptotic proteins and increasing anti-apoptotic proteins, suggested that HA can attenuate TBHP-induced apoptosis. However, these effects were reversed after mitophagy inhibition (Figures 3C–E). We subsequently detected senescence by SA-β-gal staining and found that proportion of SA-β-gal-positive cells was increased in TBHP treatment group. Whereas HA administration suppressed the percentage of senescent cells under conditions of oxidative stress, and these protective effects were abolished after mitophagy inhibition (Figures 3F,G).

### HA attenuates TBHP-induced ECM degradation in NPCs

**FIGURE 4:**
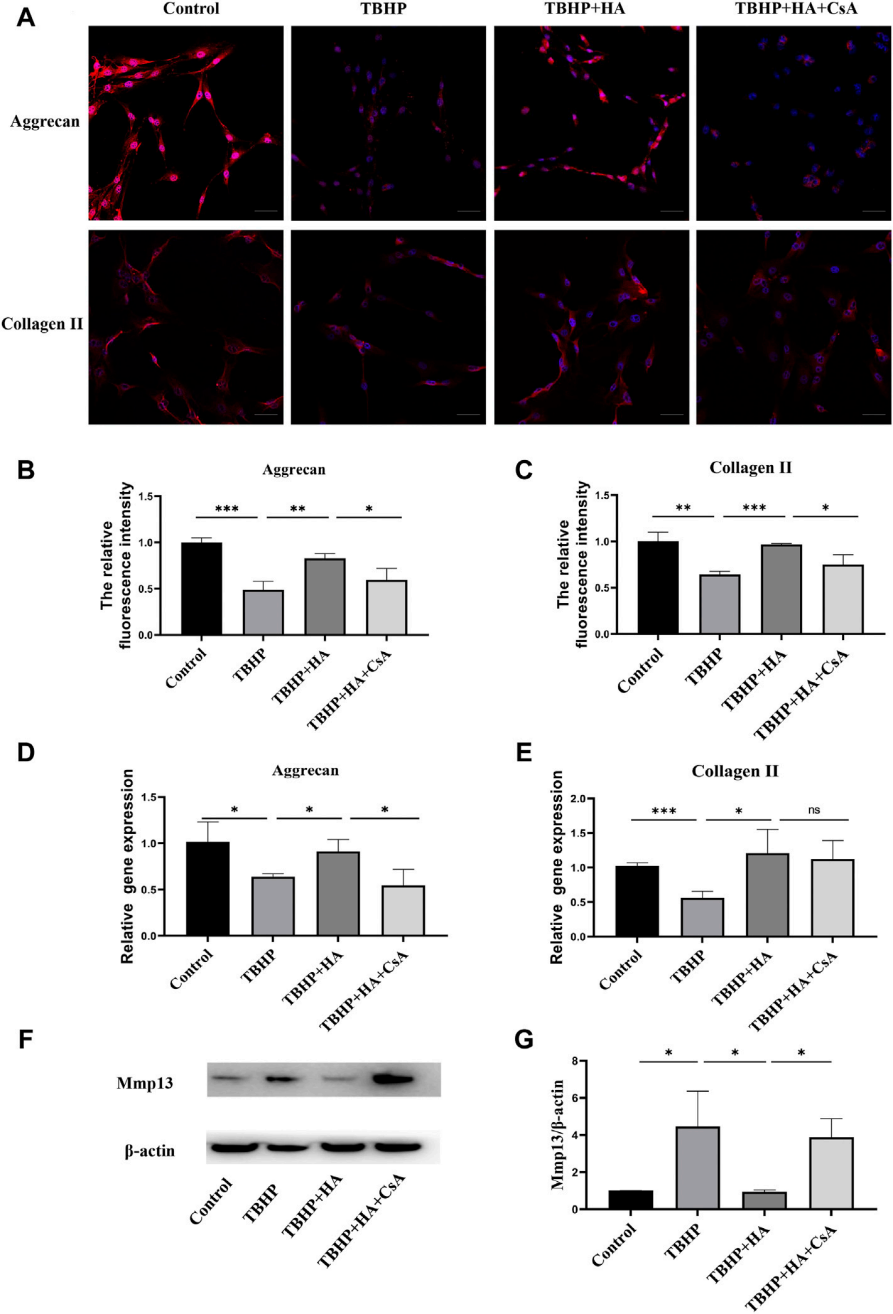
HA attenuates TBHP-induced ECM degradation in NPCs. **(A–C)** The protein expression of Aggrecan and Collagen II was detected by the immunofluorescence staining. **(D,E)** The gene expression of Aggrecan and Collagen II was analyzed by RT-qPCR. **(F,G)** The protein expression of MMP-13 was measured by western blot assay. Scale bar: 50 μM. Data are represented as the mean ± SD. ns, not significant, *p < 0.05, **p < 0.01, ***p < 0.001. *n* = 3.

The immunofluorescence staining showed that TBHP treatment markedly reduced the protein expression of Aggrecan and Collagen II. While the decreased ECM synthesis was recovered by HA administration compared to the TBHP-treatment group, and the preservation of ECM synthesis failed after mitophagy inhibition (Figures 4A–C). The gene expression of Aggrecan and Collagen II was also investigated by RT-qPCR and these results were consistent with the immunofluorescence results (Figures 4D,E). The results of the Western blot analysis showed TBHP treatment increased the expression of ECM degradation enzymes MMP-13, and the effect of HA against TBHP-induced ECM degradation was blocked after mitophagy inhibition (Figures 4F,G). Therefore, these results suggested that HA attenuates TBHP-induced ECM degradation by promoting mitophagy.

### The effect of C1QBP on HA protecting mitochondrion function under oxidative stress

**FIGURE 5:**
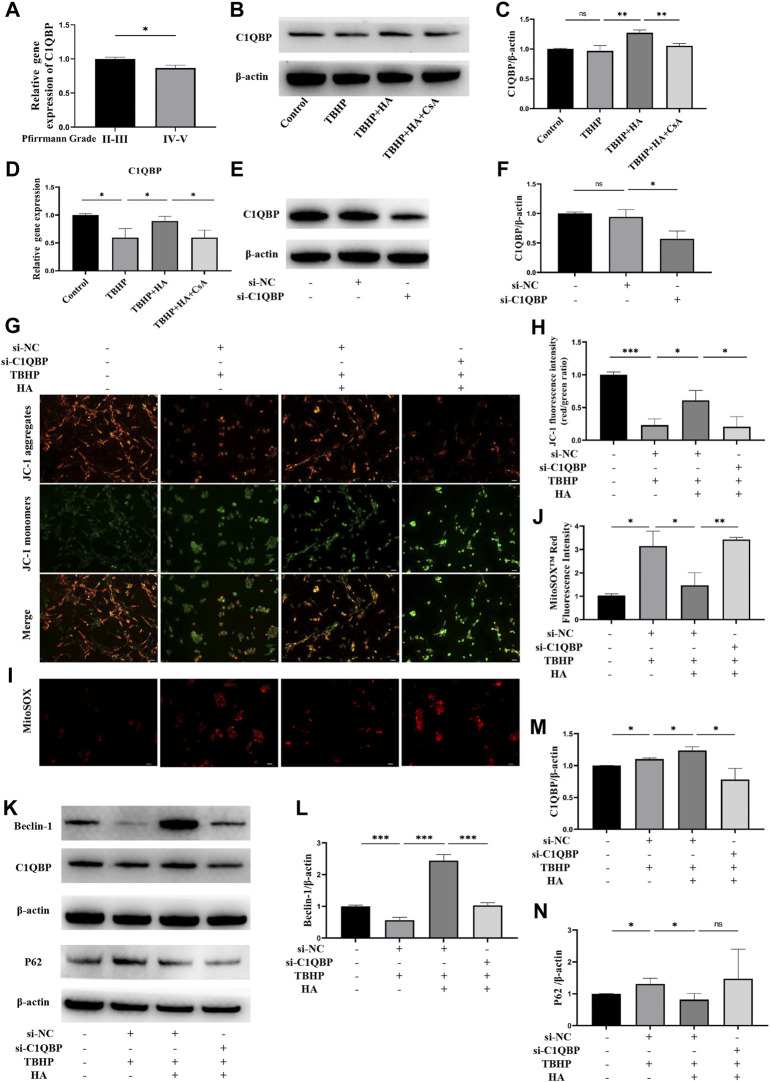
The effect of C1QBP on HA protecting mitochondrion function under oxidative stress. **(A)** Human NP tissues were divided in the mild degeneration group (II-III grade) and the severe degeneration group (IV-V grade) according to the Pfirrmann grading system, the C1QBP mRNA expression between two groups was detected by RT-qPCR. **(B–D)** The NPCs were treated with HA or CsA before TBHP stimulation, the protein expression of C1QBP was detected by Western blotting, the C1QBP mRNA expression was detected by RT-qPCR. **(E,F)** The NPCs were transfected with control siRNA (si-NC) or C1QBP siRNA (si-C1QBP), the protein expression of C1QBP was detected by Western blotting. NPCs were transfected with control siRNA or C1QBP siRNA before HA and TBHP treatment. **(G,H)** Δψm was measured by JC-1 staining. **(I,J)** The production of mitochondrial Superoxide was measured by using MitoSOX™ Red Superoxide Indicator. **(K–N)** The protein expression of Beclin-1, C1QBP and P62 was detected by Western blotting. Data are represented as the mean ± SD. ns, not significant, *p < 0.05, **p < 0.01, ***p < 0.001. Scale bar: 50 μm *n* = 3.

To determine whether C1QBP expression was correlated with IDD, human NP tissues were harvested and divided into two groups according to the Pfirrmann grading system: the mild degeneration group (II-III grade) and the severe degeneration group (IV-V grade), the expression level of C1QBP was measured by RT-qPCR. As shown in Figure 5A, we found that the expression level of C1QBP progressively decreased from the mild degeneration to severe degeneration group. We subsequently detected the expression level of C1QBP under oxidative stress. The Western blot analysis and RT-qPCR results revealed that the expression of C1QBP was increased in NPCs treated with HA, and decreased by CsA pretreatment (Figures 5B–D).

To explore the effect of C1QBP on HA protecting mitochondrion function under oxidative stress in NPCs, we suppressed the expression of C1QBP by transfection with siRNA. As shown in Figures 5E,F, the expression of C1QBP was successfully inhibited. The JC-1 staining demonstrated that HA can protect the decreased Δψm induced by TBHP. However, this protective effect was eliminated when C1QBP was knockdown (Figures 5G,H). Meanwhile, the effects of HA attenuating the mitochondrial superoxide overproduction induced by TBHP were blocked after knockdown of C1QBP (Figures 5I,J). Western blotting assay was performed to detect the protein expression of Beclin-1, C1QBP and P62. When NPCs were treated with HA under oxidative stress, the expression of Beclin-1 and C1QBP was increased, the expression of P62 was decreased. However, when the C1QBP expression was knockdown by transfection of siRNA, the increased expression of Beclin-1and C1QBP was reversed, and the expression of P62 was increased, but this change was not significant (Figures 5K–N).

### HA ameliorates NPCs apoptosis and ECM degradation in organ culture model

**FIGURE 6:**
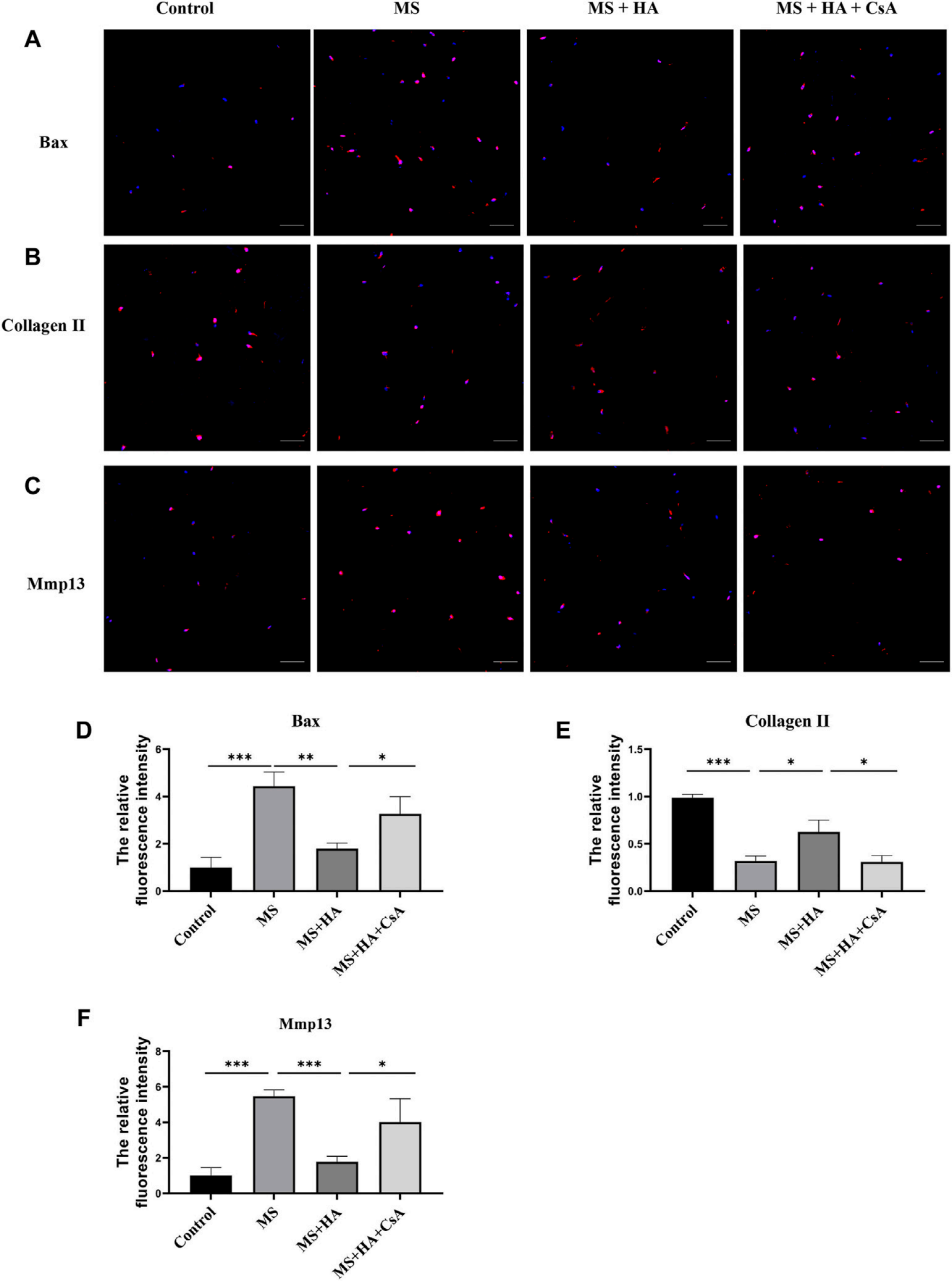
HA ameliorates NPCs apoptosis and ECM degradation in organ culture model. In organ culture model, bovine IVDs were received one strike loading and injected HA or CsA. **(A–D)** The protein expression of Bax was detected by the immunofluorescence staining. **(B–E)** The protein expression of Collagen II was detected by the immunofluorescence staining. **(C–F)** The protein expression of Mmp13 was detected by the immunofluorescence staining. Data are represented as the mean ± SD. *p* < 0.05, **p < 0.01, ***p < 0.001. Scale bar: 50 μm *n* = 3.

To confirm the protective effect of mitophagy activation by HA in IVDs, bovine IVDs were received one strike loading to induce mechanical stress, and injected HA or CsA. The immunofluorescence staining results showed that MS can promote the expression of pro-apoptotic protein Bax (Figures 6A,D) and ECM degradation enzyme Mmp13 (Figures 6C,F). While this change was attenuated by HA treatment, and CsA can reverse these effects. Moreover, the collagen II expression was decreased in MS treatment group. After treatment with HA, the collagen II levels increased markedly compared with that of the MS-treated group, but CsA can eliminate this protective effect of HA (Figures 6B,E).

## Discussion

In the current study, we found that HA treatment can induce mitophagy in NPCs, which prevented ROS overproduction and mitochondrial impairment, and subsequently inhibited cell apoptosis, senescence and ECM degradation *via* the C1QBP signaling pathway under oxidative stress conditions. Furthermore, the protective effects of HA against MS induced NPCs apoptosis and ECM degradation *via* mitophagy activation were confirmed in an IVD organ culture model.

Numerous studies have confirmed that oxidative stress plays a crucial role in the occurrence and progression of IDD (Wang et al., 2016). Mitochondrial dysfunction induced by oxidative stress is considered to be one of the important mechanisms that trigger the intrinsic pathway of NPCs apoptosis and senescence (Zhang et al., 2018). In this study, the administration of TBHP, a well-recognized exogenous inducer for oxidative stress, was demonstrated to decrease Δψm and ATP production, and cause ROS overproduction, indicating that mitochondrial function was impaired. Meanwhile, TBHP significantly increased apoptosis and senescence of NPCs, and furtherly caused ECM degradation, suggesting that oxidative stress was a common pathological factor for IDD. In addition, the expression of LC3 and Beclin-1, two key essential proteins for mitophagy initiation, was increased after TBHP treatment. It could be speculated that mitophagy was activated to remove damaged mitochondria and promote cell survival as an early response during cellular stress (Kubli et al., 2012; Xiao et al., 2017).

Hyaluronic acid is an important component of ECM and a biodegradable polymer. Its’ biological effects were independent of the molecular weight. Low MW-HA promotes the production of inflammatory mediators. Whereas, high MW-HA suppresses the production of pro-inflammatory mediators (Tavianatou et al., 2019). Our previous study also demonstrated that high MW-HA can promote the synthesis of ECM under hypoxic conditions of the intervertebral disc, suggesting that high MW-HA might be a promising therapeutic target to retard disc degeneration (Zhang et al., 2021). High MW-HA based hydrogels have been drawing increasing attention as the ideal candidates for IDD, because of their potential to repair and regenerate the NP (Zheng et al., 2021). In the present study, we found that high MW-HA pretreatment can alleviate TBHP-induced mitochondrial dysfunction, apoptosis, cell senescence and ECM degradation. Moreover, we found that high MW-HA can protect the decreased Δψm and ATP production, suggesting that HA can protect mitochondrial function from oxidative stress. These results are accordance with other reports that improving mitochondrial function under oxidative stress may be an important therapeutic mechanism for the role of HA in osteoarthritis (Grishko et al., 2009).However, how HA protects NPCs mitochondrial function remains unclear.

Mitophagy, a special type of autophagy, is essential for timely and effectively clearance of damaged mitochondria, and plays a crucial role in preserving of high-quality mitochondria and maintaining mitochondrial homeostasis (Frank et al., 2012). Increasing evidence suggests that mitophagy is a protective mechanism under pathological conditions (Chen et al., 2020). In the present study, after HA treatment, the co-localization of LC3 and mitochondria marker Tom20 was observed, and the expression of LC3 and Beclin-1 was increased, indicating that the mitophagosomes formatted and mitophagy initiation was activated. Inhibition of mitophagy by pretreatment of CsA, an inhibitor of the mitochondrial permeability transition that promotes PINK1 degradation, eliminated the protective effects of HA. These results suggest that HA can protect NPCs against mitochondrial dysfunction, apoptosis, senescence, and ECM degradation under oxidative stress by promoting mitophagy activation.

The protein C1QBP, also known as p32, receptor for globular head domains of complement 1q (gC1qR) and HABP1, is primarily localized in the mitochondrial matrix, and associated with several matrix proteins (Ghebrehiwet et al., 2019). C1QBP plays an important role in mitochondrial quality control and autophagic flux regulation under physiological conditions (Jiao et al., 2015; Noh et al., 2020). In the present study, we discovered that the expression of C1QBP in clinical NP samples decreased with the development of IDD, whereas HA upregulated the expression of C1QBP. To confirm the role of C1QBP in HA promoting mitophagy, C1QBP expression was knockdown by transfection of siRNA. We found that HA attenuated the Δψm decline and mitochondrial superoxide overproduction induced by TBHP, but this protective effect of HA on mitochondrial function can be abolished by C1QBP knockdown. Meanwhile, the expression of p62 was increased after TBHP treatment, suggesting that the autophagy flux was blocked. Blockage of autophagy flux may lead to impair the normal function of autophagy and mitochondrial homeostasis. We found that HA can down-regulate the expression of p62, but this change can be abolished by C1QBP knockdown. These results suggested that HA promotes mitochondrial dynamics and regulates mitophagy in NPCs by enhancing C1QBP. CD44, as the most widely distributed cell surface receptor recognized for HA binding, affects both cell survival pathways as well as apoptotic pathways (Knudson et al., 2004). Moreover, it was reported that HA promoted NP regeneration by enhancing the expression of CD44 and binding to CD44 in NPCs (Isa et al., 2015). Therefore, the possible mechanism of HA enhancing C1QBP expression and promoting mitophagy was through interaction with CD44 receptors.

Mechanical stress is considered as a significant risk factor for the development of IDD (Schultz et al., 2009).Moreover, our previous study also demonstrated that MS caused mitochondrial ROS accumulation and impaired mitochondrial function, which resulting in programmed NPCs death and ECM degradation in IVDs (Li et al., 2021). To confirm the protective effects of HA against NPCs apoptosis and ECM degradation induced by MS *via* mitophagy activation, one-strike loading model was used. This model has been confirmed that can reveal the pathogenesis of MS induced IDD at the early stage and suit for identify therapeutic targets for early intervention (Zhou et al., 2021). Our results showed that MS induced NPCs apoptosis by elevating Bax levels. Collagen II, as the important ECM component, plays an essential role in the physiological function and maintenance of discs. We found that the collagen II level was reduced and the expression of matrix-degradation enzyme Mmp13 was increased in bovine caudal NP tissues after MS. This finding meant that MS can induce ECM degradation. However, HA pretreatment can ameliorate NPCs apoptosis and inhibit ECM degradation. Moreover, these beneficial effects disappeared when mitophagy was blocked by CsA treatment. These results suggested that HA can mitigate MS induced disc degeneration by mitophagy activation.

There were several limitations in this study. Firstly, in the study, HA concentration of 1 μmol/L was selected, because most therapeutic pharmaceutical formulations were administered at this concentration (Rayahin et al., 2015), which may be not the optimal concentration to induce mitophagy in NPCs. Secondly, we confirmed that C1QBP played an important role in HA promoting mitophagy, but the precise molecular mechanisms need be furtherly elucidated. Thirdly, the *ex vivo* IVD organ culture model and one-strike loading model used in our study were subjected to acute mechanical stress, which is non-physiological and not reflective of IDD mechanism. Therefore, whether the research findings can be applied to other factors caused IDD needs furtherly investigation. Moreover, we identified the therapeutic effect of HA in an IVD organ culture model at the early stage, but the long-term effect needs be investigated in the future study.

## Conclusion

Our study reveals that HA treatment can induce mitophagy by enhancing C1QBP, which conferred against oxidative stress-caused ROS overproduction, mitochondrial impairment, cell apoptosis, senescence and ECM degradation. Moreover, we found that HA can ameliorate mechanical stress-caused cell apoptosis and ECM degradation by mitophagy activation. These findings help us better understand the protective role of HA in maintaining NPCs mitochondrial homeostasis, and provide more substantial evidence for the clinical applications of HA in the prevention and treatment of IDD.

## Data Availability

The original contributions presented in the study are included in the article/supplementary material, further inquiries can be directed to the corresponding authors.
